# Recent advances in lung organoid development and applications in disease modeling

**DOI:** 10.1172/JCI170500

**Published:** 2023-11-15

**Authors:** Ana I. Vazquez-Armendariz, Purushothama Rao Tata

**Affiliations:** 1 University of Bonn, Transdisciplinary Research Area Life and Health, Organoid Biology, Life & Medical Sciences Institute, Bonn, Germany.; 2 Department of Medicine V, Cardio-Pulmonary Institute, Universities of Giessen and Marburg Lung Center, Member of the German Center for Lung Research and Institute for Lung Health, Giessen, Germany.; 3 Department of Cell Biology, Duke University School of Medicine, Durham, North Carolina, USA.; 4 Duke Cancer Institute, Duke University, Durham, North Carolina, USA.; 5 Duke Regeneration Center, Duke University School of Medicine, Durham, North Carolina, USA.

## Abstract

Over the last decade, several organoid models have evolved to acquire increasing cellular, structural, and functional complexity. Advanced lung organoid platforms derived from various sources, including adult, fetal, and induced pluripotent stem cells, have now been generated, which more closely mimic the cellular architecture found within the airways and alveoli. In this regard, the establishment of novel protocols with optimized stem cell isolation and culture conditions has given rise to an array of models able to study key cellular and molecular players involved in lung injury and repair. In addition, introduction of other nonepithelial cellular components, such as immune, mesenchymal, and endothelial cells, and employment of novel precision gene editing tools have further broadened the range of applications for these systems by providing a microenvironment and/or phenotype closer to the desired in vivo scenario. Thus, these developments in organoid technology have enhanced our ability to model various aspects of lung biology, including pathogenesis of diseases such as chronic obstructive pulmonary disease, pulmonary fibrosis, cystic fibrosis, and infectious disease and host-microbe interactions, in ways that are often difficult to undertake using only in vivo models. In this Review, we summarize the latest developments in lung organoid technology and their applicability for disease modeling and outline their strengths, drawbacks, and potential avenues for future development.

## Introduction

The lung is a highly complex organ composed of over 50 identified cell types derived from the three germ layers. Such cellular diversity is distributed across various compartments, such as the airways, blood and lymphatic vessels, connective tissue, nerves, mesothelium, and lung parenchyma (1–3). Within the lung, epithelial cells are found in direct contact with the inhaled air, and among these are multiple, region-specific stem cell and progenitor populations organized along the proximal-distal axis. These include basal cells, club cells, bronchoalveolar stem cells (BASCs), and alveolar type 2 epithelial cells (AEC2s) (4–7). In the airways, basal and club cells have the capacity to self-renew and give rise to other specialized airway cells such as club, ciliated, and goblet cells upon different types of injury (8–10). In mice, BASCs are located at the bronchoalveolar duct junctions (BADJ) and can differentiate into airway and alveolar lineages depending on the injury type and severity (11, 12). The alveoli are the most distal structures of the respiratory tract; here, AEC2s are the main stem cell source. Notably, AEC2s differentiate into alveolar type 1 epithelial cells (AEC1s), which are essential for gas exchange and lung repair after injury (13, 14).

Organoids are 3D culture systems derived from stem cells that have the capacity to proliferate and differentiate into structures that resemble certain structural, biological, and functional features of the organ of origin (15, 16). Currently, Matrigel matrix is the main basement membrane used for organoid culture (17). However, Matrigel is a mouse-derived matrix, and its components are not well defined, making it difficult to translate findings into a clinical setting (18). Current alternatives are hydrogel-based matrices generated from natural (e.g., fibrin, collagen, hyaluronic acid) or synthetic materials (19–22).

**Figure 1:**
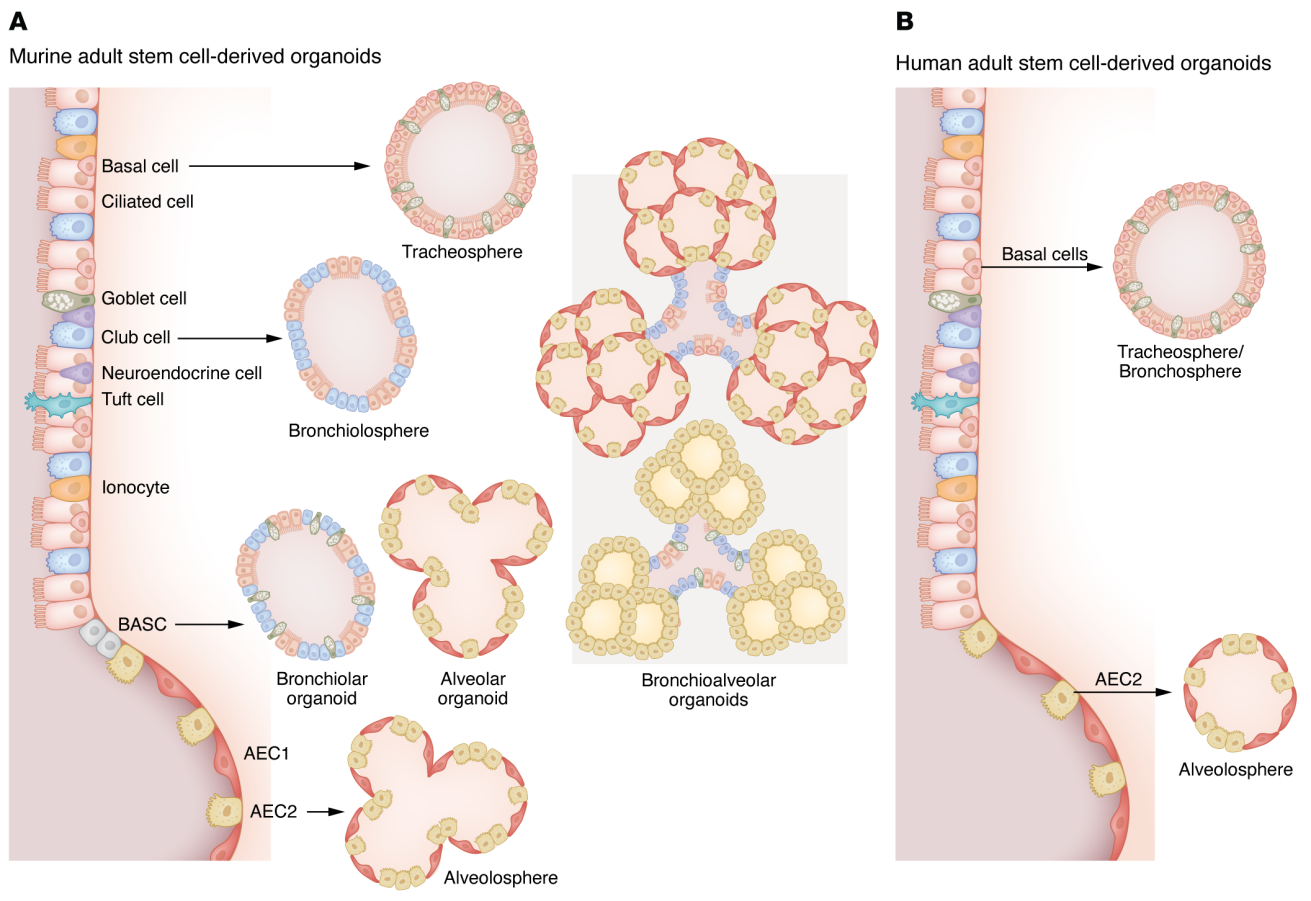
Lung organoid models derived from adult mouse and human stem cells. Several epithelial progenitor/stem cells located along the bronchoalveolar compartment of murine and human lungs are capable of generating organoids. (**A**) Murine models include organoids derived from basal cells that form tracheospheres containing basal, ciliated, and secretory cells (10, 30). Club cells can be used to develop bronchiolospheres containing club and ciliated cells (38, 39). Coculture of BASCs with lung mesenchymal cells can give rise to bronchoalveolar lung organoids (BALOs) containing tubular-like structures with basal, club, goblet, and ciliated cells and saccular-like structures composed of differentiated AEC1s and AEC2s (26). When cocultured with lung endothelial cells, BASCs can form alveolar organoids, bronchiolar organoids, and bronchiolar organoids (41). Lastly, cocultures of AEC2s with PDGFRα^+^ mesenchymal cells lead to the formation of alveolospheres, containing AEC1s and AEC2s (13, 45, 47, 111). (**B**) Lung organoids derived from human adult stem cells can be generated from basal cells and AEC2s. Basal cells can form either tracheospheres or bronchospheres, depending on their location in the airways (10, 30–33). AEC2s form alveolar-like organoids when cocultured with feeder cells and display a similar composition to their mouse counterparts (13, 14, 50).

**Figure 2:**
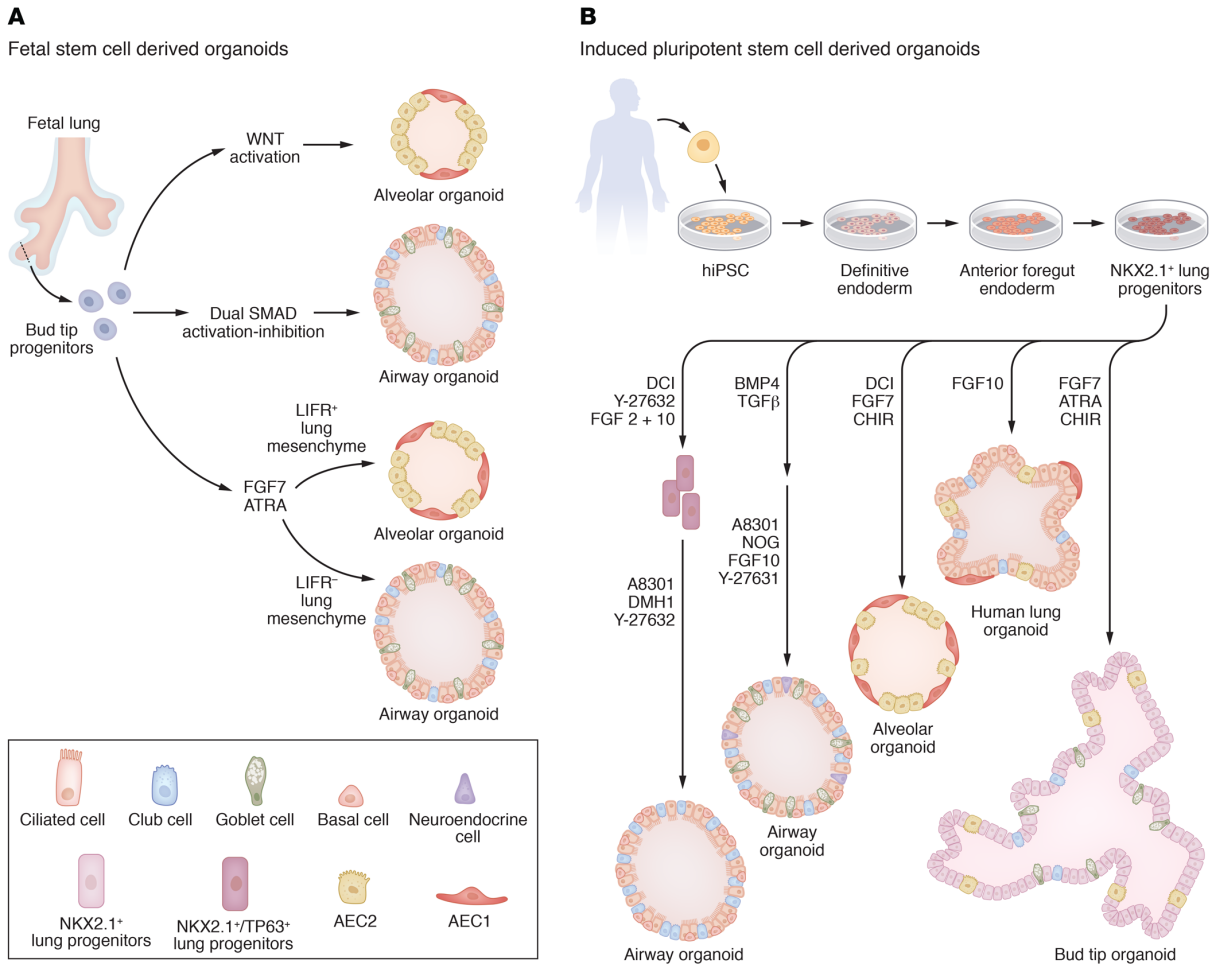
Lung organoid models derived from fetal and induced pluripotent stem cells. Schematic representation of fetal stem cells and iPSC-derived lung organoids. (**A**) Bud tip progenitor cells obtained from fetal lung tissue can be differentiated into alveolar and airway organoids. Activation of WNT pathway signaling leads to the formation of alveolar organoids containing SFTPC^+^HOPX^+^ AECs, while dual SMAD activation and inhibition lead to the development of airway organoids composed of basal, club, goblet, and multiciliated cells (24, 51). Stimulation of bud tip progenitors with ATRA and FGF-7 gives rise to alveolar-like or airway-like organoids depending on the coculture with either LIFR^+^ or LIFR^–^ lung mesenchymal cells (52). (**B**) Lung organoids derived from iPSCs are generally generated from NKX2-1 lung progenitor cells. For this purpose, iPSCs are differentiated into definitive endoderm and polarized into anterior foregut endoderm before being differentiated into lung progenitors. Dual SMAD activation and inhibition of NKX2-1 or organoid-derived NKX2-1^+^TP63^+^ progenitor cells result in the formation of airway organoids containing basal, club, goblet, and multiciliated cells and, in some conditions, SYN^+^ neuroendocrine cells (27, 28). In contrast, stimulation of lung progenitor cells with DCI, FGF, and CHIR leads to the formation of alveolar organoids comprising AEC1s and AEC2s (27, 61). In contrast, lung organoids comprising both alveolar-like cell types and airway-like cell types can be generated by addition of FGF-10 (25). Stimulation of lung progenitor cells with ATRA, FGF-7, and CHIR results in the generation of bud tip organoids containing NKX2-1^+^ lung progenitor cells, AEC2s, and club and goblet cells (25).

Lung organoid platforms have become powerful tools for modeling lung physiology and disease (Figures 1 and 2) (10, 13, 23–25). Thus far, three different types of lung organoids have been described based on the source of their starting material: (a) adult lung stem/progenitor stem cells (AdSCs), (b) fetal lung stem cells (FSCs), and (c) induced pluripotent stem cells (iPSCs). The use of different stem cell sources and optimized culture conditions such as media supplements and coculture with other relevant cell types have enabled derivation of different cell types and long-term maintenance (Tables 1 and 2) (26–28). In addition, advances in techniques such as CRISPR/Cas9–based precision gene editing have further amplified the utility of organoid models for genetic perturbations to study lung development and disease-relevant genes for personalized medicine (29). Lung organoid models have also provided valuable insights into the mechanisms that control self-renewal, survival, and differentiation potential of these epithelial populations (26–30).

Although these systems hold great promise for advancing our understanding of lung biology and disease, there are still many challenges, including efficient derivation of terminal cellular maturity and structural complexity, as well as developing standardized workflows for scalability and reproducibility. Here, we outline the most recent developments in lung organoid systems and their implications for disease modeling that could set the stage for further breakthroughs in this exciting and rapidly evolving field.

## Optimization of culture media components and cell composition

Lung organoids derived from AdSCs are attractive models for investigating epithelial stem cell potential and cellular interactions during homeostasis and disease. Both murine and human AdSC-derived organoid models have been developed from several different stem cells present along the bronchoalveolar epithelium (Figure 1) and can therefore be used to recreate specific lung environments.

Depending on the site of stem cell isolation, human basal cells can form organoids called tracheospheres (trachea), bronchospheres (large airways), or nasospheres (nasal epithelium) comprising basal, club, ciliated, and goblet cells (31–33). Tracheospheres containing basal cells and secretory and ciliated cells have also been generated from isolated murine basal cells (10). These airway models have been used to provide insights into epithelial development and signaling. For instance, SMAD and NOTCH signaling have been widely implicated in the regulation of airway stem cell proliferation and differentiation (34, 35). Distinct media supplements have been shown to modulate SMAD/BMP and NOTCH signaling during AdSC-derived organoid generation by either promoting basal cell proliferation or driving cell differentiation toward secretory and ciliated cells (30, 32, 36, 37).

Moreover, several studies have now shown that AdSC-derived organoids can model crosstalk between stem cells and their microenvironment (38–40). For instance, coculture of club cells and mesenchyme subpopulations has been used to assess mesenchymal cells’ ability to support epithelial stem cell potential. Under these conditions, cocultures gave rise to organoids called bronchiolospheres containing club and ciliated cells and revealed a distinct mesenchymal cell subset driving stem cell growth and differentiation (38, 39). Bronchiolospheres lack basal or goblet cells and are more suitable for the study of club and ciliated cell biology (39).

In 2005, BASCs were first identified at the BADJ as stem cells resistant to bronchiolar and alveolar injury in vivo (11). BASCs were found to coexpress bronchiolar and alveolar cell markers (surfactant protein C [SFTPC] and secretoglobin family 1A member 1 [SCGB1A1], respectively) and possessed self-renewal and multipotency capacities in vitro, suggesting a potential BASC contribution to club and alveolar cell maintenance (11). In line with these findings, Lee and colleagues developed a coculture system using BASCs and lung endothelial cells that gave rise to alveolar, bronchiolar, and bronchoalveolar organoids (41). In this study, the endothelium-derived BMP4/NFATc1/thrombospondin-1 (TSP1) signaling axis increased BASC proliferation and differentiation toward alveolar phenotypes. Notably, these bronchioalveolar organoids consisted of AEC2, club, ciliated, and goblet cells, but AEC1s were absent (41). Direct evidence of BASCs’ multilineage differentiation and contribution to bronchioalveolar repair after naphthalene-, bleomycin-, and influenza virus–induced injury was recently obtained using lineage tracing approaches that allowed selective labeling of SFTPC^+^SCGB1A1^+^ BASCs in the mouse lung (12, 42). Moreover, BASC coculture with resident mesenchymal cells generated complex bronchoalveolar lung organoids (BALOs) (26). These organoids were characterized by formation of tubular airway-like regions containing mature basal, secretory, and multiciliated cells, whereas distal alveolar-like areas comprised differentiated AEC1s and AEC2s capable of producing surfactant (26).

Our understanding of alveolar biology has also improved through organoids derived from murine and human AEC2s that were first described by Barkauskas et al. in 2013 (13). Using in vivo and in vitro experiments, this study revealed AEC2s as the main stem cell of the alveoli. In this organoid model, coculture of AEC2s with PDGFRα^+^ mesenchymal cells led to the formation of alveolospheres containing AEC2s and AEC1s (13). Since then, multiple organoid models using AEC2 subsets and mesenchymal cell subpopulations have been established to investigate alveolar stem cell niche interactions (43–46). More recently, alveolospheres have been used to investigate the effect of BMP signaling on AEC2 proliferation. In this setting, activation of BMP led to the reduction of AEC2 proliferation and increased AEC1 differentiation, while its inhibition promoted AEC2 self-renewal, suggesting that BMP modulation is crucial for alveolar niche maintenance (47).

Additional efforts have been made to improve the lifespan and cell differentiation of organoids derived from patients’ stem cells. Sachs et al. developed a long-term human airway organoid model from human bronchoalveolar lavage and resection material. Through modulation of TGF-β, FGF, and WNT signaling, airway organoids derived from basal cells were shown to contain basal, ciliated, and secretory/club cells that could be maintained for over a year (48). Their protocol was further optimized by Noggin (NOG) removal and DAPT/BMP4 addition into the standard airway organoid medium. Under these conditions, airway organoids generated from nasal inferior turbinate brush samples yielded significantly higher numbers of ciliated cells and could be used to model primary ciliary dyskinesia (49). In the alveoli, characterization of the WNT-responsive alveolar epithelial progenitor (AEP) subset in mice led to discovery of TM4SF1 as a surface marker for human AEP cells (14). Interestingly, TM4SF1^+^ AEPs isolated from human samples gave rise to functional alveolospheres when cocultured with a human fetal lung fibroblast cell line (14). In addition, Tran and colleagues created a human AEC2 immortalized cell line using SV40 large T antigen lentiviral transfection and Y-27632 (a RHO/ROCK pathway inhibitor) media supplementation. After initial 2-dimensional expansion, AEC2s cocultured with a mouse lung fibroblast cell line formed alveolospheres expressing AEC1 and AEC2 markers (50).

Innovations in organoid derivation protocols have led to the use of FSC-derived lung organoids for studies on lineage specification and cellular interactions during development and disease (Figure 2A). In 2017, Nikolić and colleagues developed organoids from human and mouse fetal lung bud tips (LBTs) (24). LBT-derived organoids were grown in medium containing seven factors known to modulate crucial signaling pathways involved in lung morphogenesis, including growth factors (EGF, FGF-7, and FGF-10), BMP signaling inhibitors (NOG and SB431542), and WNT signaling activators (RSPO1 and CHIR) (24). Through this combination, organoids retained expression of the lung-specific transcription factor NK2 homeobox 1 (NKX2-1) and coexpressed lung progenitor markers SRY-box transcription factor 2 (SOX2) and SOX9 but did not contain differentiated cell types. Commercial human airway medium was then used to drive differentiation within LBT-derived organoids toward more specialized bronchiolar lineages such as goblet, basal, and ciliated cells (24). Conversely, derivation of LBT organoids into alveolar lineages was partially achieved through coculture with freshly sorted human lung mesenchyme in medium containing FGF-7, FGF-10, CHIR, DCI (a combination of dexamethasone, cAMP, and the intracellular cAMP activator IBMX), triiodo-l-thyronine (T3), and the NOTCH inhibitor DAPT (24). These settings resulted in the formation of organoids containing AECs coexpressing SFTPC and homeodomain-only protein (HOPX) (24). Single-cell RNA sequencing (scRNA-Seq) analysis of LBT organoids also identified SMAD signaling as a major cue for airway patterning during lung development (51). In this system, SMAD activation was promoted by addition of TGF-β and BMP4 during the first days of culture followed by prolonged SMAD inhibition through A8301 and NOG addition (51). This dual signaling modulation led to the formation of proximal airway organoids composed of clonal basal cells capable of self-renewal and multilineage differentiation into basal-, goblet-, club-, and ciliated-like cells (51).

Besides FSC-derived organoids’ utility in elucidating cell fate decisions, these models can also be valuable tools to uncover novel mesenchymal-epithelial cell interactions during lung development. Transcriptional and spatial profiling of human developing lungs recently revealed a distinct mesenchymal cell population in the LBT that was enriched for the WNT agonist R-Spondin 2 (RSPO2) (52). When cocultivated with isolated LBT-derived epithelial cells in media containing FGF-7 and ATRA but not CHIR, LIFR^+^ (RSPO2^+^) mesenchyme induced organoid proliferation and alveolar lineage derivation into SFTPC^+^ and RAGE^+^ AECs (52). Alternatively, coculture with LIFR^–^ mesenchyme increased airway marker secretoglobin family 3A member 2 (SCGB3A2) expression, implying that RSPO2^+^ mesenchyme provides a niche signal for distal bud tip progenitor maintenance and multipotency by supporting WNT signaling activation (52). In line with these findings, NOTUM, a WNT inhibitor, was expressed in actin alpha 2–positive (ACTA2^+^) myofibroblasts in the distal LBT (53). Coculture of NOTUM-overexpressing fibroblasts and alveolar-like LBT organoids led to loss of SFTPC expression, suggesting that NOTUM^+^ myofibroblasts control alveolar cell fate during lung development by blocking WNT signaling activation in the distal tip epithelium (53). These recent studies not only uncovered novel mesenchymal-epithelial crosstalk during lung morphogenesis but also provide insights into how these systems can be further directed to mimic developmental processes in vitro.

The establishment of novel protocols to generate human iPSC–derived lung organoids represents a major advance in pulmonary disease modeling, drug screening, and regenerative medicine (Figure 2B). Generally, through addition of specific factors into the culture media, murine and human iPSCs are first directed toward definitive endoderm, followed by sequential generation of anterior foregut endoderm (AFE), ventral anterior foregut endoderm cells (VAFECs), and, lastly, NKX2-1^+^ lung progenitors (54–56). Coculture of an SFTPC-GFP reporter human iPSC cell line and human fetal lung mesenchyme showed for the first time that carboxypeptidase M–positive (CPM^+^) surface marker could be employed to isolate lung progenitors from VAFECs (57). Notably, organoids derived from VAFECs contained NKX2-1– and CPM-coexpressing cells, as well as AEC1s and AEC2s (57). Accordingly, Konishi and colleagues used CPM as a surface marker for NKX2-1^+^ VAFEC isolation. Formed organoids could be driven toward airway-like organoids comprising multiciliated cells by addition of DAPT into the medium (58).

A primary objective of the subsequent investigations with iPSCs has been to optimize media conditions to promote airway and alveolar cell maturity. In line with this goal, gene editing of human iPSC cell lines using TALENs or CRISPR/Cas9 technologies enabled more detailed characterization and isolation of NKX2-1 lung progenitors (28, 29). Airway organoids containing functional basal cells were achieved using a novel NKX2-1^GFP^/tumor protein P63 (TP63)^tom^ dual fluorescent reporter iPSC line (28). Initially, NKX2-1 lung progenitors were purified based on GFP expression and cultured in medium containing FGF-2, FGF-10, DCI, and the RHO/ROCK pathway inhibitor Y-27632 (28).

After a month in culture, NKX2-1^+^TP63^+^ cells were isolated and seeded in commercially available PneumaCult-Ex Plus medium (Stemcell Technologies) along with SMAD and ROCK inhibitors. Formed organoids contained basal cells that showed clonal expansion and multilineage differentiation into basal-, club-, and ciliated-like cells when cultured in air-liquid interface conditions (28). Nonetheless, the need for reporter lines for stem cell isolation limits the system applicability and translation into clinical scenarios. In the mentioned study, the use of fluorescent reporters could be replaced by surface expression of CD47, CD26, nerve growth factor receptor (NGFR), and epithelial cell adhesion molecule (EPCAM) cell markers, facilitating prospective basal cell isolation from patient-specific iPSCs for clinical applications (28).

In a comparative study, cells solely isolated based on CPM expression contained more NKX2-1^+^ progenitors than cells expressing CD47 and/or CD26, indicating that further optimization of culture conditions is necessary to obtain the desired cellular differentiation (59). In this regard, similar studies developed human alveolospheres by combining NKX2-1 reporter iPSC lines and media composition optimization (60, 61). For instance, culture of sorted NKX2-1–GFP^+^ cells in CHIR, FGF-10, and FGF-7 media followed by addition of DCI gave rise to organoids coexpressing NKX2-1 and AEC2 markers (29). Coculture of NKX2-1^+^ cells with distal embryonic mesenchyme resulted in higher levels of SFTPC expression, suggesting that distal fibroblasts induce alveolar differentiation during tip specification (29). In another related study, human iPSC derivation toward organoids with AEC lineages was directed by preconditioning of NKX2-1^+^ VAFECs with medium containing CHIR, FGF-10, FGF-7, and DAPT. This treatment led VAFECs to express higher levels of CPM, which facilitated isolation and coculture of CPM^hi^ cells with fetal mesenchymal cells that give rise to organoids comprising AEC2s and AEC1s (60). Notably, fibroblast-free alveolar organoids with AEC2s could be obtained by DCI, FGF-7, CHIR, Y-27632, and SB431542 media supplementation (60).

Moreover, based on previous studies, NKX2-1^GFP^SFTPC^tom^ dual reporter iPSC lines were first cultured in medium containing CHIR, BMP4, and ATRA (a combination called CBRa) to acquire NKX2-1^GFP^ progenitors while FGF-7, CHIR, and DCI supplementation was used for subsequent NKX2-1^GFP^SFTPC^tom^ alveolar organoid formation (61, 62). Nevertheless, this culture setting has substantial contamination by gastric cells (~20%), likely due to early presence of mid- and hind-gut cells (63, 64). Lastly, airway organoids derived from NKX2-1^GFP^ cells were developed by withdrawal of CHIR from the medium, further supporting other studies indicating that WNT signaling activation promotes AEC2 proliferation while suppressing proximal lineage differentiation (65, 66). While combination of reporter iPSC lines and standardization of media components represents a valuable strategy for organoid formation and cell lineage specification, upcoming iPSC-derived lung organoid systems should be generated without the need of reporter cell lines to broaden these models’ clinical applicability.

While iPSC-derived AEC2s have the advantage of high throughput and scalability over AdSCs, the latter cells denote age-associated maturity and maintain genetic and epigenetic characteristics of the donor or patient lungs. Over the years, efforts have been made to comparatively assess the self-renewal, maturity, and differentiation capacity of iPSC-derived and primary AEC2s in ex vivo models (67, 68). In the case of iPSC-derived AEC2s, inhibition of WNT signaling using XAV939 (tankyrase inhibitor) led to AEC1 differentiation (68, 69). However, WNT withdrawal from the culture medium was not sufficient to induce AEC1 differentiation of both iPSC-derived and primary AEC2s (68, 70). Studies in mice have revealed that YAP signaling is highly enriched in AEC1s and ectopic activation of YAP maintains AEC1 cell identity and is sufficient to promote AEC2 to AEC1 differentiation (71–76). To test whether activation of YAP signaling can promote iPSC-derived AEC2 differentiation, Burgess and colleagues used both genetic and pharmacological activation approaches. Indeed, these studies revealed that activation of YAP signaling was sufficient to induce AEC1 gene expression in iPSC-derived AEC2s (77). Such studies are still required to assess FSC-derived AEC2 potential in organoid cultures. In the case of AdSCs, addition of human serum induced AEC2 to AEC1 differentiation (43). However, the specific components of the human serum capable of inducing AEC2 differentiation are not known, suggesting that identification of such molecules may have therapeutic value for both cell replacement and regenerative therapies.

Current human iPSC models contain epithelial cells found on the alveolar or airway lung compartments, but only a few systems feature proximal to distal patterning. In this regard, SOX9^+^SOX2^+^ lung bud organoids (LBOs) that resemble LBT cellular composition were generated after induction of VAFECs in the presence of FGF-10, FGF-7, and CBRa (78). Although LBOs did not exhibit mature airway cells or AEC1s, they contained goblet and club cells in the proximal structures and AEC2s in the distal tips (78). In another study, Miller and colleagues developed human lung organoids (HLOs) and bud tip organoids (BTOs) from iPSC-derived foregut spheroids (25). HLOs were created by culture in high levels of FGF-10 and included TP63^+^ and FOXJ1^+^ airway-like epithelium surrounded by a diffuse network of mesenchymal cells and epithelial cells coexpressing SFTPC and HOPX alveolar markers. Conversely, lung progenitors cultured in FGF-7, CHIR, and ATRA developed BTOs with SOX2^+^, mucin 5AC–positive (MUC5AC^+^), and SCGB1A1^+^ airway-like regions and SOX2^+^, SOX9^+^, SFTPC^+^, and ID2^+^ bud tip–like domains (25). In a follow-up protocol, NKX2-1^+^ bud tip progenitor–like cells coexpressing SOX9, SOX2, and CPM were enriched to promote organoid formation. Following digestion, cell sorting, and culture of NKX2-1^GFP^–expressing cells, bud tip progenitor–like cells expanded, generating spheroids with a robust NKX2-1 expression (27). Using previously published protocols, these spheroids could be efficiently directed toward alveolar or airway organoids (51, 61). Nonetheless, additional culture optimization is still necessary to establish human iPSC–derived organoids that more accurately recapitulate the lung architecture.

## Innovative biological insights provided by organoid gene editing

Incorporation of novel genetic tools into organoid systems is transforming the field of regenerative medicine and disease modeling (79). Genetic tools such as CRISPR/Cas9 and transgenic approaches have enabled precise gene editing and study of specific gene functions in lung organoids. Deletion of the transcription factor grainyhead-like 2 (*GRHL2*) in primary human basal cells followed by airway organoid culture identified GRHL2 as a molecular regulator of barrier function and ciliated cell differentiation (80). Further, genetic modification of LBT progenitor cells uncovered NKX2-1 as a key WNT modulator. In this study, NKX2-1 overexpression supported development of alveolar-like organoids, while NKX2-1 deletion or CHIR media withdrawal promoted airway organoid formation (53). These data indicated that NKX2-1 is a key downstream regulator of WNT signaling driving alveolar differentiation while also inhibiting airway lineage specification.

Numerous studies have used CRISPR-based gene editing for loss and gain of function, as well as for generation of reporters and ectopic activation of oncogenes in iPSCs (28, 59, 61, 66, 81–89). However, the loss of specific genes may impair iPSCs’ ability to survive, replicate, or differentiate in culture. In the case of AdSCs, delivery of transgenes via electroporation or viruses also has challenges, as, in many cases, these techniques have not been well established or are not efficient. AdSCs may also activate stress pathways and lose their ability to self-renew (90, 91). Therefore, more efforts are needed to optimize and benchmark methods for delivery of multiple DNA constructs of varying sizes, achieve efficient gene editing, and determine robust cellular markers to assess cell identities after gene manipulation. Recent studies have made some progress in delivering large constructs via lentiviruses into FSCs. To avoid toxicity associated with ectopically expressed Cas9 and its fusion proteins, Sun et al. used a two-layered control system to tightly regulate transgene expression (92). The authors used this system for conditional knockdown and gain of function of genes in FSC-derived lung progenitors using CRISPR interference and CRISPR activation technologies, respectively. Further, Sun et al. have also developed strategies (Easytag workflow) to insert transgenes expressing fluorescent proteins in human FSC–derived lung progenitors (92). These reporters allowed them to purify specific progenitors and assess their potential ex vivo in organoid cultures. In the future, this promising methodology could potentially be used to enhance the efficiency and specificity of gene editing techniques and ensure organoid model reproducibility.

## Generation of complex lung organoid systems

Even though recent developments in organoid technology have improved our ability to mimic the structural complexity of the bronchoalveolar comportment, current human lung organoid models still lack terminal cellular maturity, particularly mature alveolar lineages, and progressive branching formation. In recent studies, human iPSCs and mathematical methods have been employed to identify the regulatory networks responsible for early embryonic tissue patterning (93, 94). Ideally, integration of experimental data and computational predictions could be used to accelerate culture optimization and achieve organoid branching morphogenesis and terminal differentiation.

Human organoid platforms are currently limited to nonimmune cell types found in the lung and can only be vascularized through transplantation into mice. In the murine system, the bronchoalveolar lung organoid (BALO) model allowed the direct addition of alveolar macrophages into the lung organoids. Notably, scRNA-Seq analysis of these organoids revealed a significant upregulation of genes involved in epithelial cell differentiation when supplemented with macrophages (26). Therefore, the generation of an immune-competent human organoid model, for example by direct cell microinjection, could allow study of the interactions between immune cells and various other cellular components ex vivo.

Additionally, recent protocols generating iPSC-derived endothelial cells might allow the generation of vascularized iPSC-derived lung organoids that could more fully reiterate lung complexity (95, 96). A challenge in incorporating other tissues such as endothelial, immune, and mesenchymal cells is to optimize culture conditions to also support nonepithelial tissues. One approach could be to coax iPSCs to codevelop both endoderm-derived epithelial cells and other tissues from the mesoderm. Such approaches have been successfully used in liver and intestinal organoids (89, 97–103). Further, Workman and colleagues also generated neural crest–derived neurons that were integrated into intestinal organoids (104). Such models could expand the utility of lung organoids to model interactions with neuronal cells. For integrating multiple tissues into organoids, one must also consider the cellular sources for each of these tissue components. Studies have shown that fibroblasts from one area may alter the properties of epithelial cells in another region in coculture experiments (105). Therefore, it is important to use age-matched and region-specific resident counterparts as opposed to cell lines (e.g., MRC5, 3T3 cells). For instance, recent studies have used iPSCs derived from a mouse line that harbors a fluorescent reporter driven by a lung mesenchyme–specific enhancer in the *Tbx4* gene (106, 107). Using these cells, the authors generated and purified mouse lung mesenchymal cells from iPSCs and used them for coculture with epithelial cells (108). By modulating growth conditions, the authors were able to produce mesenchymal cells that could induce proximal or distal lung fates as well as maturation of epithelial cells in cocultures (108). Interestingly, the presence of mesenchymal cells expressing Tbx4 has been detected in LBOs, which could be a valuable approach to study epithelial-mesenchymal cell crosstalk during homeostasis and disease (63). These studies provide a proof of concept that organoids with different cell combinations can be developed and they can potentially be used for complex disease modeling.

## Studying lung disease and regeneration using organoid models

Much of our current understanding about lung repair after injury primarily comes from mouse models (109). Technical advances and the availability of genetically modified mouse models have facilitated the analysis of different region-specific stem and progenitor cells in lung repair after injury (13, 30). Despite these advantages, mouse lungs do not fully recapitulate the complexity and the cellular diversity of human lungs. In this context, organoid models have been valuable in understanding human-specific cell-cell interactions and cell fate choices during regeneration. The scalability of organoids has also enabled identification of pathways that can sustain and enhance self-renewal potential of human airway basal cells and AEC2s (30, 44, 47). Tadokoro et al. performed a chemical screen and found that inhibition of both TGF-β and BMP enhanced basal cell self-renewal in organoid cultures, whereas activation of IL-6 promoted basal cell differentiation toward ciliated cells at the expense of club cells (30, 37). Similarly, other studies also performed growth factor and cytokine screens on murine AEC2s and found that treatment with IL-1β and TNF-α enhanced both AEC2 replication and colony-forming efficiency. Interestingly, these findings directly mirrored the results from in vivo studies (110, 111). Recent studies have also used scRNA-Seq of AEC2 organoids to uncover a transitional state during AEC2 to AEC1 differentiation (70). These intermediate states have been implicated in pulmonary fibrosis, since transitional AEC accumulation has been associated with disease pathogenesis (112). Moreover, another study revealed that stimulation of AEC2s with IL-1α could trigger the induction of alveolar transitional states via the HIF-1α/glucose pathway (113).

Recent developments in single-cell technologies have facilitated the discovery of multiple known, rare, and unknown cell populations in the human lung (1, 114). For example, such studies have identified a previously uncharacterized SCGB3A2 cell population in human terminal and respiratory bronchioles, a cell type that has not been found in mouse lungs (70, 115, 116). Intriguingly, scRNA-Seq studies have found that these cells are either enriched or depleted in pulmonary fibrosis and chronic obstructive pulmonary disease (COPD), respectively (70, 115, 116). Therefore, understanding the mechanisms that control the development and maintenance of SCGB3A2^+^ cells is of substantial interest. These findings also highlight the challenges associated with the use of animal models owing to species differences and emphasize the need to develop human-based ex vivo lung models (70, 115, 116). To overcome this obstacle, two studies have adapted previously established iPSC- and AdSC-based culture models to assess cell fate potential and/or cellular origin of SCGB3A2^+^ cells (43, 70, 116, 117). Basil and colleagues used iPSC-derived distal lung progenitors that contain a fluorescent reporter under the control of the SCGB3A2 promoter (116). This reporter enabled purification of SCGB3A2^+^ cells and demonstrated that these cells can generate AEC2s in organoid cultures (116). The authors have made similar observations with SCGB3A2^+^ cells purified directly from human distal airways using CD66c, a cell surface marker highly enriched in these cells (116). In contrast, another study by Kadur Lakshminarasimha Murthy and colleagues found that AEC2s can convert to SCGB3A2^+^ cells in organoid cultures and de novo in human lungs after injury and in diseases including idiopathic pulmonary fibrosis and COPD (70). Interestingly, this study also proposed an approach in which AEC2-derived SCGB3A2^+^ cells contribute to ectopic bronchiolar structures found in idiopathic pulmonary fibrosis. Notably, these two groups used similar computational cell trajectory prediction approaches but found opposing lineage relationships, which were validated using organoid-based models (70, 116). Therefore, future studies need to focus on developing in vivo lineage tracing models to assess the lineage relationships of SCGB3A2^+^ cells in their native tissues at homeostasis, in repair after injury, and in diseases.

In addition, organoid models have been used to study lung tumorigenesis. Genetically modified cells isolated from mouse models and human iPSC–derived progenitors as well as human lung tumor–derived cells have been used to generate lung tumor models. Interestingly, these models recapitulate morphological, histological, and genetic profiles similar to native tumors (118, 119). Among the different systems, human pluripotent stem cell– and iPSC-derived models have been at the forefront with respect to their use in disease modeling. Airway organoids derived from several stem cell sources have been extensively used to study different classes of cystic fibrosis–associated mutations, including both common (e.g., F508) and rare (W1282X and G542X), and the associated phenotypes (48, 66, 83–87). Further, organoid models also revealed the ability of airway cells to reconstitute functional CFTR after correcting the mutations or after pharmacological modulation (83–87). While the current gold standard is to use air-liquid interface models for assaying CFTR activity, multiple groups have optimized forskolin-induced swelling assays using airway organoid cultures (83). Besides cystic fibrosis, lung organoid models have been used to study genetic mutations associated with other lung diseases including primary ciliary dyskinesia, Hermansky-Pudlak syndrome, and pediatric and adult interstitial lung diseases (28, 49, 59, 61, 78, 81, 82, 120). Among AdSC-derived models, airway organoids are more commonly used than alveolar organoids to study disease pathogenesis. Therefore, future studies need to focus on developing and optimizing methods to apply gene editing and disease modeling of adult alveolar organoid models. Taken together, these studies further highlight the utility of organoid models to study normal and disease lung physiology ex vivo.

## Applications of organoid models to study infectious biology

Organoid models have been serving as excellent tools to study lung infectious diseases. Despite their use in various biological contexts, it was not until the COVID-19 pandemic that lung organoid technology became more regularly employed in the infectious biology field (121–123). During this time, it was recognized that the SARS-CoV-2 virus did not infect standard animal models, and safety, ethics, and cumbersome experimental procedures limited the utility of large-animal models for this disease.

Before the pandemic, multiple cell lines, including A549, Calu-3, VeroE6, and Caco-2, had been used for viral propagation and for studying virus–host cell interactions, viral cell tropism, and viral life cycle. Although these cell lines show high susceptibility for infection, their intrinsic abnormalities make them unsuitable for studying viral biology. For example, SARS-CoV-2 enters human host cells via the ACE2 receptor, but the commonly used cell line VeroE6 uses the endosomal pathway to infect the cells. Further, studies have shown that such alternate entry mechanisms induce adaptive mutations in SARS-CoV-2, which make them less transmissible and pathogenic (124, 125).

Since the pandemic, the field has swiftly recognized the importance of using species-, organ-, or tissue-specific cell types to study viral pathogenesis. In this regard, both iPSC- and AdSC-derived organoid models have been shown to recapitulate the route of viral entry, viral propagation, and the mechanisms of viral shedding (43, 126–131). Multiple studies have demonstrated the presence of SARS-CoV-2 entry factors (e.g., ACE2) as well as processing enzymes (e.g., TMPRSS2) in lung epithelial cells, including airway secretory cells, ciliated cells, and AEC2s (43, 130, 132). Further, using a reverse genetics system approach to generate a GFP reporter virus, infection efficiency was tested in different regions along the respiratory tract (133).

Together with organoid modeling and SARS-CoV-2 entry factor expression analysis, this study revealed a proximal-distal infection gradient along the upper and lower respiratory tract (133). Additionally, multiple studies showed that SARS-CoV-2–infected cells in alveolar organoids exhibit gene expression programs similar to those seen in COVID-19 autopsy samples (43, 134–136). This includes elevated levels of various proinflammatory chemokines (CXCL10, CXCL11, and CXCL17), interferon pathway components (type I and III IFNs), cell death pathway components (TNFSF10, CASP1, CASP4, CASP5, and CASP7), and downregulation of surfactant-related genes (SFTPC, SFTPD) (43, 128, 130).

Further, Katsura et al. found that treatment of alveolospheres with IFNs recapitulates features of virus infection, including cell death. In contrast, alveolospheres pretreated with low-dose IFNs show a reduction in viral replication, suggesting the prophylactic effectiveness of IFNs against SARS-CoV-2 infection (111). In addition to viral pathogenesis, recent studies performed to identify novel targets against SARS-CoV-2 have used lung organoid models to screen and test therapeutic small molecules (127, 137).

In summary, organoid models have provided new insights into COVID-19 disease pathogenesis and in principle can be used for screening and identifying therapeutic compounds. Besides SARS-CoV-2, organoid models have been used to study other pathogens, such as respiratory syncytial virus (RSV), parainfluenza virus, measles, Lloviu virus, and enterovirus (78, 123, 131, 138–142). In the case of RSV, organoids derived from multiple sources including human FSC– and human iPSC–derived and adult airway models have been applied to study infection as well as cellular responses (48, 123, 141). Notably, these studies allowed the examination of several biological processes, including airway epithelial cellular remodeling, cellular motility, immune cell recruitment, cytoskeletal rearrangements, apical protrusion, and syncytia formation similar to cytological phenotypes observed in patients (48, 143).

## Future directions

Despite lung organoid models’ ability to mimic cell fate choices and cellular programs, these systems have intrinsic limitations in fully recapitulating the cell maturity, cell-to-cell interactions, and multicellular complexity observed in vivo. For instance, long-term maintenance of alveolar organoids derived from human AdSCs with differentiated AECs, particularly AEC1s, is still critically missing. Future studies should focus on developing culture conditions that promote differentiated AEC1 survival. In addition, although BASC-derived murine organoids display high structural complexity, future research aiming at the identification of BASC equivalents in humans could allow the generation of more complex organoids derived from patients’ samples. Lastly, further combination of optimized culture settings, genome editing tools, and scRNA-Seq analyses could improve the generation of tailored lung organoid systems for disease modeling and drug discovery. Overall, the current state of organoid technologies indicates that these platforms have been and will remain a valuable resource in the search for novel cellular and molecular players involved in the development, progression, and/or resolution of respiratory illnesses.

## Figures and Tables

**Table 2:**
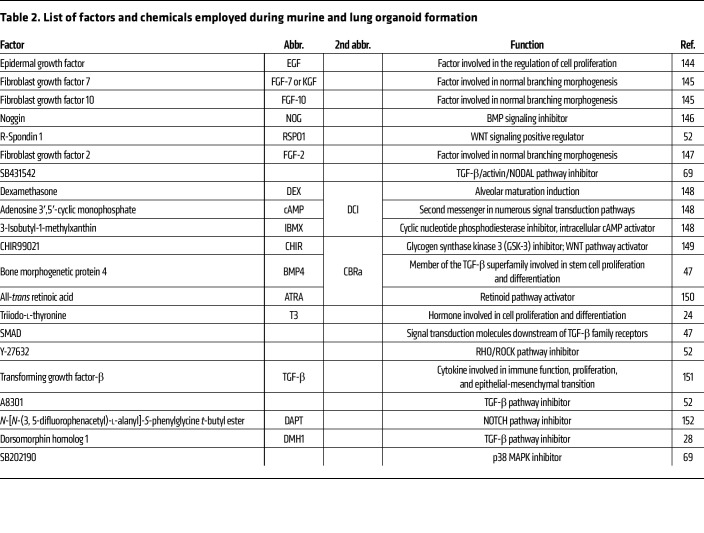
List of factors and chemicals employed during murine and lung organoid formation

**Table 1:**
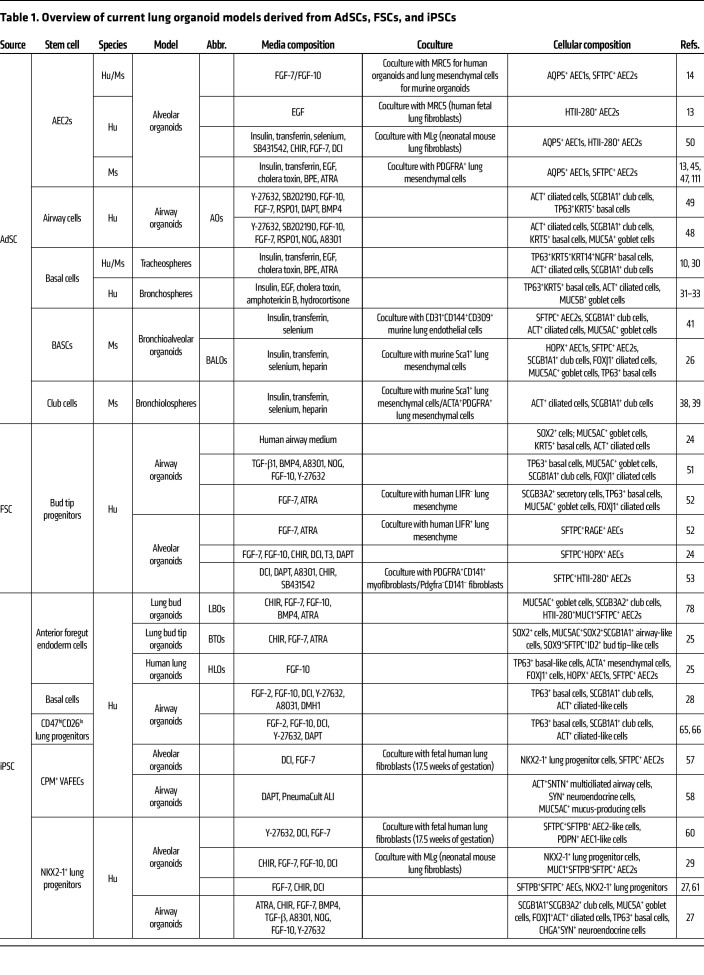
Overview of current lung organoid models derived from AdSCs, FSCs, and iPSCs

## Version 1 11/15/2023

Electronic publication
